# Different Expression of Thyroid-Specific Proteins in Thyroid Cancer Cells between 2-Dimensional (2D) and 3-Dimensional (3D) Culture Environment

**DOI:** 10.3390/cells11223559

**Published:** 2022-11-10

**Authors:** Ji Min Oh, Prakash Gangadaran, Ramya Lakshmi Rajendran, Chae Moon Hong, Jaetae Lee, Byeong-Cheol Ahn

**Affiliations:** 1 Department of Nuclear Medicine, School of Medicine, Kyungpook National University, Daegu 41944, Korea; 2 BK21 FOUR KNU Convergence Educational Program of Biomedical Sciences for Creative Future Talents, Department of Biomedical Science, School of Medicine, Kyungpook National University, Daegu 41944, Korea; 3 Department of Nuclear Medicine, Kyungpook National University Hospital, Daegu 41944, Korea

**Keywords:** thyroid cancers, 3-dimensional culture, Thyroid differentiation, Hypoxia

## Abstract

The two-dimensional (2D) monolayer culture as a conventional method has been widely applied in molecular biology fields, but it has limited capability to recapitulate real cell environments, being prone to misinterpretation with poor prediction of in vivo behavior. Recently, the three-dimensional (3D) spheroid culture has been studied extensively. Spheroids are self-assembled cell aggregates that have biomimicry capabilities. The behavior of thyroid cancer under the 3D spheroid culture environment has been studied; however, there are no reports regarding differences in the degree of thyroid cancer cell differentiation under 2D and 3D culture conditions. This study investigated the expression of thyroid differentiation proteins related to iodide-metabolizing mechanisms in thyroid cancer cells under different culture conditions. Four thyroid cancer cell lines and one thyroid follicular epithelial cell line were grown in adherent 2D cell culture and 3D spheroid culture with agarose-coated plates. We observed changes in proliferation, hypoxia, extracellular matrix (ECM), cytoskeleton, thyroid-specific proteins, and thyroid transcription factors. All cell lines were successfully established in the spheroid following cell aggregation. Proliferation considerably decreased, while hypoxia-inducible factor 1-α(HIF1-α) was promoted in 3D spheroids; moreover, 3D spheroids with thyroid cancers showed diminished thyroid differentiation markers, but thyroid follicular epithelial cells revealed either a maintenance or weak decline of protein expression. We verified that the 3D spheroid culture environment can be similar to in vivo conditions because of its alterations in numerous cellular and functional activities, including morphology, cellular proliferation, viability, hypoxia, ECM, cytoskeleton, and thyroid differentiation, compared to the conventional 2D monolayer culture environment. An in vitro experimental study using 3D spheroid culture is ideal for the faster discovery of new drugs.

## 1. Introduction

Thyroid cancer is the most common cancer of the endocrine system, and its incidence rate has been continuously increasing worldwide [1]. Most thyroid cancers are the differentiated thyroid cancer (DTC) type, which have an excellent prognosis with multimodal treatment, including thyroidectomy followed by adjuvant radioactive iodine (RAI) therapy and thyroid-stimulating hormone (TSH) suppressive therapy [2]. Nevertheless, local recurrence and distant metastasis inevitably occur in some patients with DTC, and some of these recurrences have characteristics of dedifferentiation (i.e., RAI refractoriness and poor prognosis) [1]. Lenvatinib and sorafenib, which are multitargeted tyrosine kinase inhibitors, are the current treatment options as systemic therapy for these patients [3]. Although these drugs have therapeutic effects for RAI-refectory thyroid cancers, the threats of severe adverse effects and eventual resistance remain [4]. Therefore, many ongoing preclinical studies aim to determine effective drug candidates that target various signaling pathways related to the pathogenesis of thyroid cancers.

Many drug candidates that show promising effects in vitro with cancer cells proceed to in vivo studies with animal models (mainly mice) for evaluating their effects within living organisms [5,6]. However, the results of these studies usually differ from those of in vitro studies [7,8]; thus, the accuracy of in vitro studies for predicting the efficacy of in vivo studies has remained controversial and remarkably delays the success of drug development [8]. Drug efficacy in vitro is usually different because it is a two-dimensional (2D) cell culture condition, which simulates an environment wherein cells grow as a monolayer depending on culture plates or flasks with attachment into the plastic surface [9]. The 2D monolayer culture environment lacks the ability to recapitulate the real tumor microenvironment of cell-to-cell or cell-to-extracellular matrix (ECM) interactions. This had different gene expression and signaling pathways compared to in vivo environments [7,10,11,12]. Because the culture environment might influence in vitro studies for excavating redifferentiation drugs in thyroid cancer, efficacy and molecular mechanisms can be evaluated more accurately by using in vitro experimental environments similar to the natural environment of cancer growth.

Originally, multicellular spheroids (MCSs) were fabricated by Moscona and Moscona through a self-assembly process of cell suspensions. These tissue-like aggregates could acquire characteristics similar to those of original tissues [13]. These MCSs developed under three-dimensional (3D) culture conditions can bio-mimic the environment with respect to cell-to-cell or cell-to-ECM interactions, hypoxia, chemical penetration, and nutrition gradients [14,15]. To date, most studies on thyroid cancer under the 3D culture environment have focused on testing the anti-tumor effect of certain drugs [16,17,18]. A few studies have described the change in thyroid differentiation proteins using patient’s samples under the 3D culture condition [17,19,20], but no comparative study has discussed the change in differentiation characteristics of thyroid cancer cells between the 2D and 3D culture environments. A recent report emphasized that the tumor microenvironment, which involves hypoxia and quiescence, could affect the expression of the sodium iodide symporter (NIS) and membrane targeting after NIS-mediated RAI uptake in cancer cells expressing exogenous NIS [21]. Although the study involved non-thyroid cancer cells, it nevertheless suggested the possibility of a similar phenomenon in thyroid cancer by the same underlying mechanisms of impairment of NIS expression and translocation.

In the situation, we hypothesized that different experimental microenvironments of the 2D monolayer culture and 3D spheroid culture could affect the differentiation status of thyroid cancer cells with the expression of thyroid-specific proteins related to iodide-metabolizing machinery. This study aimed to evaluate the differences in thyroid cancer cells between 2D and 3D culture environments in terms of their expression of thyroid-specific proteins.

## 2. Materials and Methods

### 2.1. Cell Lines

Human anaplastic thyroid cancer cell lines (8505C, BHT101, CAL62, Hth7, and SW1736), human papillary thyroid cancer cell lines (BCPAP, BHP10-3SCp, K1, and TPC-1), and a human thyroid follicular epithelial cell line (Nthy-Ori 3-1) were prepared for the experiments. The 8505C, BCPAP, BHT101, and CAL62 were purchased from Deutsche Sammlung von Mikroorganismen and Zellkulturen (DSMZ; Braunschweig, Germany). The Nthy-Ori 3-1, Hth7, TPC-1, and SW1736 were given by Dr. Minho Shong (School of Medicine, Chungnam National University, Daejeon, Korea), while the BHP10-3SCp cells were given by Dr. Young Joo Park (School of Medicine, Seoul National University, Seoul, Korea). The 8505C and SW1736 cells were maintained in a Roswell Park Memorial Institute-1640 medium (HyClone, Logan, UT, USA) supplemented with 10% fetal bovine serum (FBS; Gibco, Grand Island, NY, USA) and 1% penicillin–streptomycin (Gibco, Carlsbad, CA, USA). The BCPAP, BHP10-3SCp, CAL62, Hth7, Nthy-Ori 3-1, and TPC-1 cells were maintained in Dulbecco’s modified Eagle’s medium (DMEM high glucose; HyClone) supplemented with 10% FBS and 1% penicillin–streptomycin. The BHT101 cell line was maintained in DMEM high glucose medium (HyClone) supplemented with 20% FBS and 1% penicillin–streptomycin in a humidified incubator at 37 °C with 5% CO_2_.

### 2.2. 2D Monolayer and 3D Spheroid Cell Culture

Standard cell culture plates were used for adherent 2D monolayer cultures. For 3D spheroid cell cultures, cell culture plates were coated with 1% agarose (Cat No. A9045, Sigma, St. Louis, MO, USA), which was diluted in double-distilled water and autoclaved, and kept in a humidified incubator (37 °C, 5% CO_2_) for 1–2 h to solidify the agarose. Then, the cells were seeded and cultured in a CO_2_ incubator at 37 °C for 1–10 days to allow for spheroid formation. To excavate the cell lines that have distinctive spheroid formation ability, 1 × 10^3^ and 5 × 10^3^ cells with 100 µL medium were seeded in 70 µL agarose-coated 96-well plates and observed every 3 days using phase microscopy (AXIO, Carl Zeiss, Oberkochen, Germany) until day 10. For 2D culture, 1 × 10^3^ cells/100 µL were seeded in 96-well plates without agarose gel coating. After confirming the spheroid formation ability, one thyroid follicular epithelial cell line (Nthy-Ori 3-1) and four thyroid cancer cell lines (8505C, BHP10-3SCp, Hth7 and SW1736) were selected for further experimentation. Afterward, 2 × 10^6^ cells/10 mL were seeded with/without agarose coating in 100 mm culture plates and the experiments were performed.

### 2.3. Morphology and Measurement of Spheroid Size

After initiating the spheroid culture on agarose-coated culture plates, images in random fields were acquired to monitor the morphology of spheroids via phase microscopy in 3-day intervals. Additionally, the sizes of spheroids diameter were measured using ZEISS ZEN imaging software (AXIO). The size of at least 20 spheroids was calculated for each cell line and represented as distribution graphs.

### 2.4. Cell Proliferation Assay

The cells (1 × 10^3^ cells/1 well) were seeded in 96-well plates and incubated in a humidified incubator at 37 °C with 5% CO_2_ from days 1 to 10 for 2D culture. For 3D culture, the cells (1 × 10^3^ or 5 × 10^3^ cells/1 well) were seeded in an agarose-coated 96-well plate and cultured in a CO_2_ incubator at 37 °C up to day 10. Following incubation until those days, cell counting kit-8 (CCK-8) reagents (Dojindo Molecular Technologies, Inc., Rockville, MD, USA) were added to the wells and incubated for 2 h. Next, absorbance was measured at 450 nm using a microplate reader (Bio-Rad Laboratories, Hercules CA, USA). To validate the accuracy of cell proliferation in the 3D culture environment, we utilized the CellTiter-Glo^®^ 3D Cell Viability Assay (Promega, Madison, WI, USA), which could detect viable cells by measuring the amount of adenosine triphosphate (ATP) present. After the maintenance of the spheroids until those days, 100 µL CellTiter-Glo^®^ 3D reagent with equal volume of cell culture medium was added into each well and mixed vigorously for 5 min to promote cell lysis, and the culture plates were incubated at room temperature (RT) for 30 min. Afterward, the luminescence signals were recorded using multimode microplate reader (TECAN, Männedorf, Switzerland). Cell viability results were represented as a percentage relative to day 1.

### 2.5. Protein Extraction and Western Blot Analysis

To harvest the cultured cells at a fixed time, cells were washed in chilled phosphate buffered saline (PBS). After removal of the PBS, the cells were exposed to Trypsin-EDTA (0.25% Trypsin with 2.25 mM EDTA) for 3 min in a humidified incubator (5% CO_2_ with 37 °C) to detach from culture plates and added the complete culture medium to inactivate Trypsin. Next, cells were centrifuged at 1500× *g* for 3 min and supernatant was removed. Then, the cell pellets were lysed using a radioimmunoprecipitation assay (RIPA) buffer (Thermo Fisher Scientific, Rockford, IL, USA) containing protease and phosphatase inhibitors (Thermo Fisher Scientific). The lysed cell pellets were briefly vortexed 3 times at 10 min intervals and subsequently centrifuged at 13,000× *g* for 20 min at 4 °C. Following centrifugation, supernatants containing the proteins were transferred to new tubes. The proteins were quantified to determine their concentration using the bicinchoninic acid (BCA) protein assay kit (Thermo Fisher Scientific). For Western blot analysis, 8–12% sodium dodecyl sulfate (SDS) polyacrylamide gels were prepared depending on the protein sizes. Equal amounts of protein (μg) were mixed with 4× Laemmli Sample Buffer (Catalog number: #161-0747Bio-Rad Laboratories), including 2-mercaptoethanol (Catalog number: #161-0710, Bio-Rad Laboratories) with a final concentration of 355 mM, and the mixtures were boiled at 95 °C for 5 min for the disruption of molecular interactions. The prepared protein samples were electrophoresed on SDS polyacrylamide gels and then transferred onto polyvinylidene fluoride membranes (Millipore, Burlington, MA, USA). The membranes were blocked with 3% bovine serum albumin (BSA, genDEPOT, Katy, TX, USA) prepared in Tris-buffered saline containing 0.05% Tween-20 (TBS-T, BIOSESANG, Seoul, Korea) for 2 h and then probed overnight at 4 °C with respective primary antibodies diluted in 0.5% BSA. After washing 3 times with TBS-T, membranes were probed with horseradish peroxidase (HRP)-conjugated secondary antibodies (Anti-Mouse IgG, HRP-linked Antibody (Cat No. #7076), Anti-Rabbit IgG, HRP-linked Antibody (Cat No. #7074)) diluted in 0.5% BSA for 1 h at RT, and then, the membranes were washed 3 times with TBS-T again. For detecting protein bands, membranes were exposed to the Amersham™ ECL Select™ Western Blotting Detection Reagent (Cytiva, Marlborough, MA, USA) and detected using a Fusion FX chemiluminescence analyzer system (Vilber Lourmat, Marne-la-Valle’e, France) as per the manufacturer’s instructions. Band intensities were quantified using a Fusion FX chemiluminescence analyzer system. Details of used antibodies are described in Scheme S1. Blot images were cropped and prepared using PowerPoint (Microsoft, Redmond, WA, USA); the contrast was adjusted, if necessary, for better visualization.

### 2.6. Hematoxylin and Eosin (H&E) Staining and Immunohistochemistry

Spheroids were harvested and fixed in 6-well plates using 4% paraformaldehyde of neutral pH (2 ml, BIOSESANG) at RT overnight. Fixed spheroids were molded into cryomold (Laborimpex, Bruxelles, Belgium) with 2% agarose gels, embedded into paraffin, cut into 5 μm sections, and mounted on slides. The specimen sections on slides were deparaffinized and stained using hematoxylin (Mirax, Kyungkido, Korea) and eosin (Mirax).

For immunohistochemistry analysis, we performed antigen retrieval procedure with 10 mM sodium citrate buffer (pH 6.0) by heating in microwave for 10 min. Next, the slides were kept at RT for 20 min and then washed using distilled water. Then, we performed the blocking of endogenous peroxidase with 3% hydrogen peroxide in methanol for 10 min and washed 3 times using PBS. Next, specimen sections were probed with primary antibody with anti-NIS (Cat No. NBP1-70342; Novus Biologicals; working dilution: 1:100), then with the anti-mouse/anti-rabbit EnVision FLEX/HRP labeled polymer (Dako, Santa Clara, CA, USA) used according to the manufacturer’s instruction. Both H&E and immunohistochemistry-stained slides were analyzed using light microscopy (Nikon, Tokyo, Japan).

### 2.7. Immunofluorescence Analysis

For the 2D monolayer culture, 5 × 10^4^ cells were seeded in 4-well chambers and cultured in a humidified incubator at 37 °C with 5% CO_2_ for 2 days. Thereafter, cells were fixed with 4% paraformaldehyde for 10 min at RT and washed 3 times with PBS for 5 min. Next, cells were permeabilized with PBS including 0.5% Triton X-100 for 5 min at RT, followed by 3 washes with PBS containing 0.05% Tween-20 (PBS-T) for 5 min.

In the case of 3D spheroid cultures, spheroids were fixed using 4% paraformaldehyde at RT overnight. After molding in 2% agarose gels, the fixed spheroids were embedded into paraffin, cut into 5 μm thickness sections, and mounted onto slides. The specimen sections on slides were deparaffinized, and then, we performed antigen retrieval with 10 mM sodium citrate buffer (pH 6.0) by heating in microwave for 10 min. Next, the slides were kept at RT for 20 min and then washed using distilled water. Then, we performed the blocking of endogenous peroxidase with 3% hydrogen peroxide in methanol for 10 min and washed 3 times using PBS.

Next, the samples from both 2D monolayer and 3D spheroid cultures were blocked using 3% BSA in PBS-T for 1 h; this was incubated with primary antibodies overnight at 4 °C. Afterward, the cells were rinsed 3 times with PBS-T for 5 min and probed with secondary antibodies for 1 h at RT. Coverslips were mounted onto slides with Vecta mounting medium containing 4′,6-diamidino-2-phenylindole (Vector Laboratories, Burlingame, CA, USA) and analyzed using confocal laser microscopy (LSM 5 exciter; Zeiss, Oberkochen, Germany). The list of used antibodies in these experiments is described in Scheme S2.

### 2.8. Statistical Analysis

All data are expressed as mean ± standard deviation (SD). Two groups of data were statistically analyzed via Student’s *t* test using the GraphPad Prism 5 software version 5.01 (GraphPad Software, Inc., La Jolla, CA, USA). Statistical significance was set at *p* values of < 0.05.

## 3. Results

### 3.1. Observation of Spheroid Morphologies from Thyroid Cancer and Thyroid Follicular Epithelial Cells

First, nine human thyroid cancer cell lines and one thyroid follicular cell line were cultured in 96-well plates coated with 1% agarose to excavate cell lines with well-established spheroid formation ability. As shown in the representative images from Supplementary Figures S1 and S2, the morphologies and initiation of aggregation for spheroid formation were different regardless of the cell type. Despite differences in the time of initiation of spheroid formation, all five cell lines (8505C, BHP10-3SCp, Hth7, SW1736, and Nthy-Ori 3-1) formed tight and almost indestructible spheroids, which shrunk in size over time under the 3D culture environment. Some cell lines (8505C and SW1736) depended on cell number to form spheroids (i.e., seeding of 1 × 10^3^ cells did not form well-established spheroids compared to 5 × 10^3^ cells). However, other thyroid cancers formed no well-established spheroids despite the high number of cells; moreover, the BHT101 and CAL62 cell lines initiated cell aggregation but did not develop into compacted spheroid shapes. Lastly, the BCPAP, K1, and TPC-1 cell lines showed no aggregation followed by failure of spheroid formations. Based on these observations, we classified the cell lines depending on the degree of spheroid formation and chose five cell lines (8505C, BHP10-3SCp, Hth7, SW1736, and Nthy-Ori 3-1) showing tight aggregation for further experiments.

### 3.2. Size Distribution of MCSs

**Figure 1:**
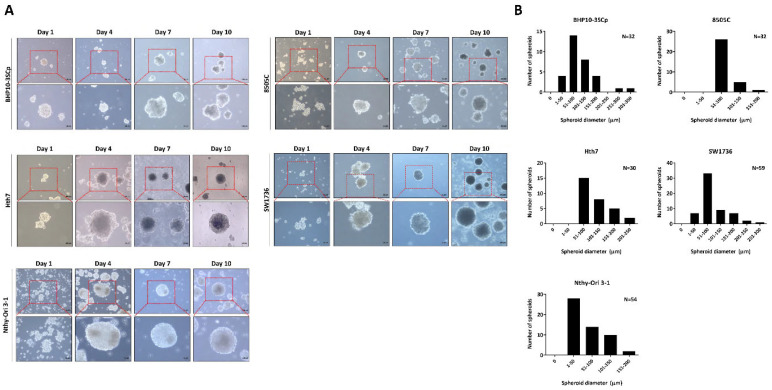
Establishment of 3D spheroids. Four thyroid cancer cell lines (BHP10-3SCp, 8505C, Hth7, and SW1736) and one thyroid follicular epithelial cell line (Nthy-Ori 3-1) were well established into spheroids with cell aggregation. (**A**) Morphology of spheroids imaged via contrast microscopy every 3 days. (**B**) Size distribution of 3D spheroids on day 7.

Next, these cell lines were seeded in 100 mm culture plates coated with 1% agarose, and their morphologies were observed once every 3 days (Figure 1A). These cell lines initiated cell aggregation; some cell lines even generated spheroids (BHP10-3SCp and Hth7). However, although the spheroids were well established on agarose-coated plates, they had variable sizes and irregular shapes, likely due to their matrix-on-top characteristics [22]. Our results suggest that five of the nine cell lines had well-established spheroids on agarose-coated plates.

Following observation of spheroids formation, we chose day 7 as the period of culture to allow for spheroid formation. Next, the 96-well plate was transferred to the 100 mm plate to harvest large amounts of spheroids. Numerous spheroids formed on the 100 mm culture plate. Additionally, both the size and shape of spheroids were irregular regardless of cell type. For the quantitative analysis of the spheroid size, we acquired images of random fields in the 3D culture. The diameter of spheroids was measured based on at least 20 spheroids, whereas their size was represented with frequency distribution graphs because their matrix-on-top characteristics caused variations in size. As shown in Figure 1B, all cell lines revealed various sizes of spheroids. The spheroids with BHP10-3SCp cells had different sizes measuring between 38.2 and 314.7 μm (median: 88.8 μm). Conversely, the diameters of spheroids with 8505C cells ranged from 54.9 to 168.2 μm (median: 82.2 μm). Spheroids with Hth7 cells had diameters ranging from 69.6 to 244.2 μm (median: 101.7 μm). The SW1736 spheroids had diameters ranging from 32.0 to 271.0 μm (median: 79.8 μm). In spheroids with Nthy-Ori 3-1 thyroid follicular cells, their diameters ranged from 59.0 to 224.4 μm (median: 97.4 μm). These results revealed that the matrix-on-top characteristic with the agarose-coated method does not yield spheroids of uniform size and shape. Furthermore, all spheroids from various cell lines had various diameters, with an overall average size of 80–100 μm on day 7.

### 3.3. ATP Levels and Cell Proliferation Capacity in the 3D Spheroids Culture Environment

**Figure 2:**
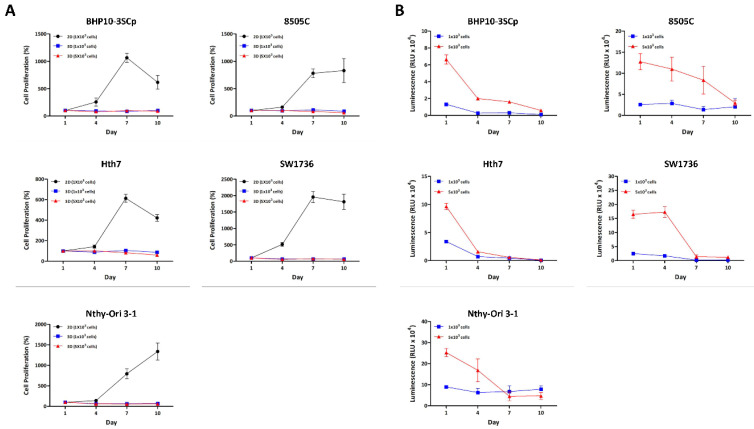
Cell proliferation between the 2D monolayer and 3D spheroid cultures. All cell lines were incubated in both 2D monolayer and 3D spheroid cultures until day 10. Cell proliferation was measured every 3 days using the CCK-8 reagent and CellTiter-Glo^®^ 3D Cell Viability Assay. (**A**) Cell proliferation between 2D monolayer culture and 3D spheroid culture with CCK-8 reagent. (**B**) Cell proliferation in 3D spheroid culture with CellTiter-Glo^®^ 3D Cell Viability Assay.

We aimed to compare the differences in ATP amount, which allows us to estimate the cell proliferation/viability capacity between 2D and 3D different culture environments over time. As shown in Figure 2A, cellular proliferation with viability was confirmed between the 2D monolayer and 3D spheroids using a CCK-8 reagent. Most cell lines in the 2D culture condition grew until day 7, but some cell lines (BHP10-3SCp, Hth7, and SW1736) had decreased cell proliferation on day 10 due to overgrowth. However, cell proliferation capacity was not increased under 3D culture conditions regardless of cell type. Additionally, we evaluated cell proliferation capacity with viability using the CellTiter-Glo^®^ 3D reagent, which is specialized for measuring ATP amounts in spheroids. Spheroids (i.e., seeded groups of 1 × 10^3^ cells) demonstrated a reduced ATP amount measured via luminescence signals on day 4 compared to day 1. However, it was not changed from day 4 to 10. This could be attributed to the loss of cell aggregation ability as shown in Supplementarys Figures S1 and S2. For the seeding of 5 × 10^3^ cells, the ATP amount sharply decreased in BHP10-3SCp and Hth7 from day 4, while this gradually decreased until day 7 in 8505C and SW1736 (Figure 2B). Additionally, the thyroid follicular epithelial cell line demonstrated drastically decreased ATP on day 7. Taken together, all cell lines had decreased ATP amounts (measured via luminescence signals) compared to day 1 with well-established spheroid formation. The cell proliferation capacity clearly decreased alongside the ATP amount compared to day 1 on 3D culture condition.

### 3.4. Histological Analysis of 3D Spheroids

**Figure 3:**
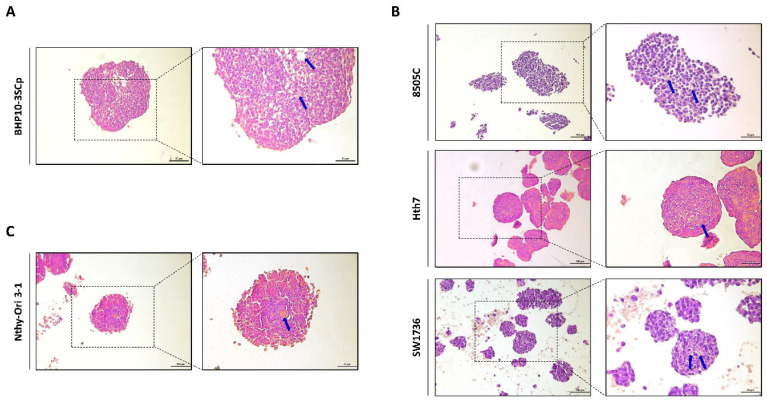
Hematoxylin and eosin (H&E) staining of 3D spheroids. Representative hematoxylin and eosin staining images. (**A**) Papillary thyroid cancer cell line. (**B**) Anaplastic thyroid cancer cell lines. (**C**) Thyroid follicular epithelial cell line. Blue arrow indicated eosin staining showing necrosis. Scale bar: 50 µm.

Next, we performed histological analysis of 3D spheroids with H&E staining. As shown in Figure 3, all cell lines with 3D culture were stained with both hematoxylin, which denotes viable cells with nuclei staining, and eosin, which stains proteins showing necrosis. However, ratio of H&E staining differed according to the composition of 3D spheroids. In BHP10-3SCp spheroids, we observed the detachment of cells and positive eosin staining in the inner layer (Figure 3A). Spheroids derived from other cell lines, including anaplastic thyroid cancer (ATC) and thyroid follicular epithelial cells, were also strongly stained with eosin in the inner layers compared to the proliferating zones (Figure 3B,C). The outer layer with proliferating zones were predominantly stained with hematoxylin staining, indicating viable cells, whereas the inner layer was likely necrotic due to a lack of oxygen and nutrients.

### 3.5. Expression of Cell Proliferation Markers between the 2D Monolayer and 3D Culture Conditions

**Figure 4:**
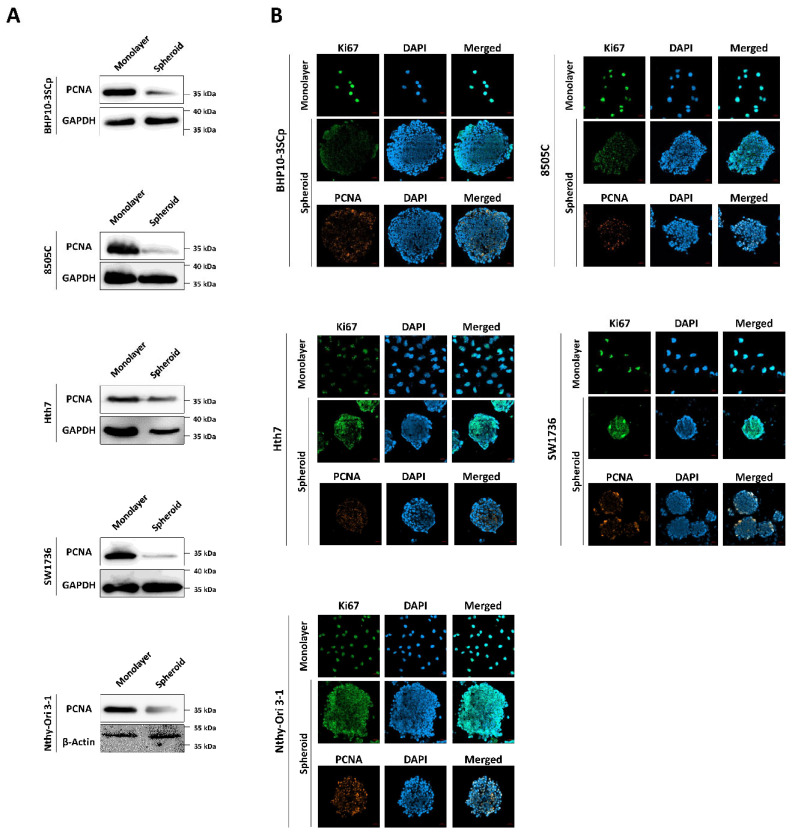
Immunofluorescence and Western blot analysis with cell proliferation markers. (**A**) Western blot analysis showing expression of PCNA, as a cell proliferation marker. GAPDH and β-Actin were used as loading control. (**B**) Representative immunofluorescence images showing cell proliferation with proliferating cell nuclear antigen (PCNA), Ki67. Scale bar: 50 µm.

Next, we confirmed the effect of the 3D spheroids’ culture on cell proliferation with markers of proliferation, such as Ki67 and proliferating cell nuclear antigen (PCNA). As shown in Figure 4A, PCNA protein expression was significantly downregulated under 3D spheroids compared to the 2D monolayer culture. Additionally, we monitored the localization of proliferation markers on cells. Both Ki67 and PCNA were strongly expressed in the nucleus in the 2D monolayer culture, regardless of cell type (Figure 4B). However, the 3D spheroid culture had different patterns compared to the 2D monolayer culture. There was a sharp decrease in the expression of proliferation markers in the inner layer compared to the outer layer.

### 3.6. Acceleration of Hypoxia under 3D Spheroid Culture Conditions

**Figure 5:**
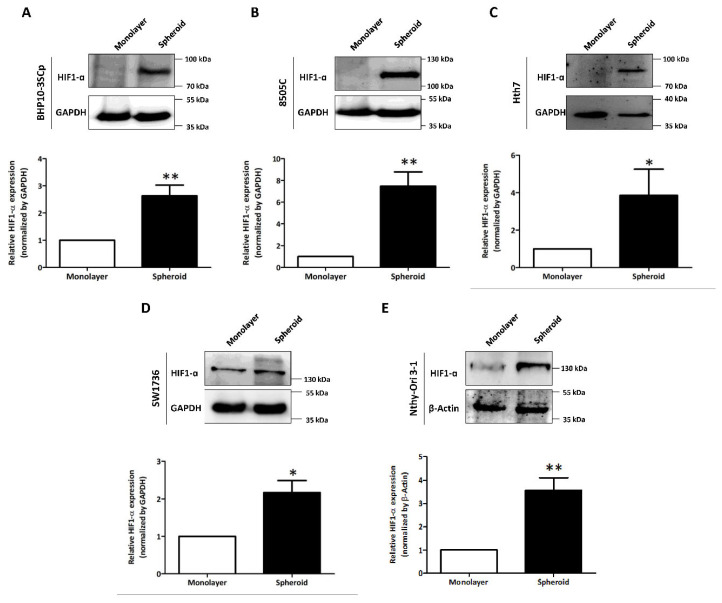
Investigation of hypoxia-inducible factor 1-α (HIF1-α) under the 2D monolayer and 3D spheroid condition. (**A**) Papillary thyroid cancer cell line (BHP10-3SCp). (**B**–**D**) Anaplastic thyroid cancer cell lines - (**B**) 8505C, (**C**), Hth7 (**D**) SW1736. (**E**) Thyroid follicular epithelial cell line (Nthy-Ori 3-1). GAPDH and β-Actin were used as loading control. A quantitative analysis displayed under the Western blot images. The mean ± standard deviation (SD) values from four independent experiments are presented. * *p* < 0.05, ** *p* < 0.01 (Student’s *t* test).

We observed and compared variable expression of hypoxia-inducible factor 1-α (HIF1-α) in the 2D monolayer and 3D spheroid cultures. As shown in Figure 5, the four thyroid cancer cell lines and one thyroid follicular epithelial cell line demonstrated accelerated HIF1-α expression in the 3D spheroid culture, but this was low/undetectable in the 2D monolayer culture. Quantitative analysis also showed the statistically significant upregulation of HIF1-α expression in the 3D spheroid cultures versus in the 2D monolayer culture (BHP10-3SCp: 2.63 ± 0.40; 8505C: 7.47 ± 1.32; Hth7: 3.85 ± 1.40; Nthy-Ori 3-1: 3.56 ± 0.55; and SW1736: 2.17 ± 0.32). Additionally, the size of HIF1-α could vary due to different forms; its theoretical molecular weight is 93 kDa, but its weight after post-translational modification is 110–130 kDa. Our results showed that only the 3D spheroids of BHP10-3SCp had HIF1-α detected between 70 and 100 kDa, indicating its theoretical size. However, the other four cell lines of 3D spheroid culture had sizes between 100 and 130 kDa, which are more indicative of the post-translation process. These results revealed that 3D spheroid culture with cell aggregation triggers hypoxia and upregulates HIF1-α regardless of cell type.

### 3.7. Evaluation of Expression of ECM and Cytoskeleton Proteins between 2D Monolayer and 3D Spheroid Culture Conditions

**Figure 6:**
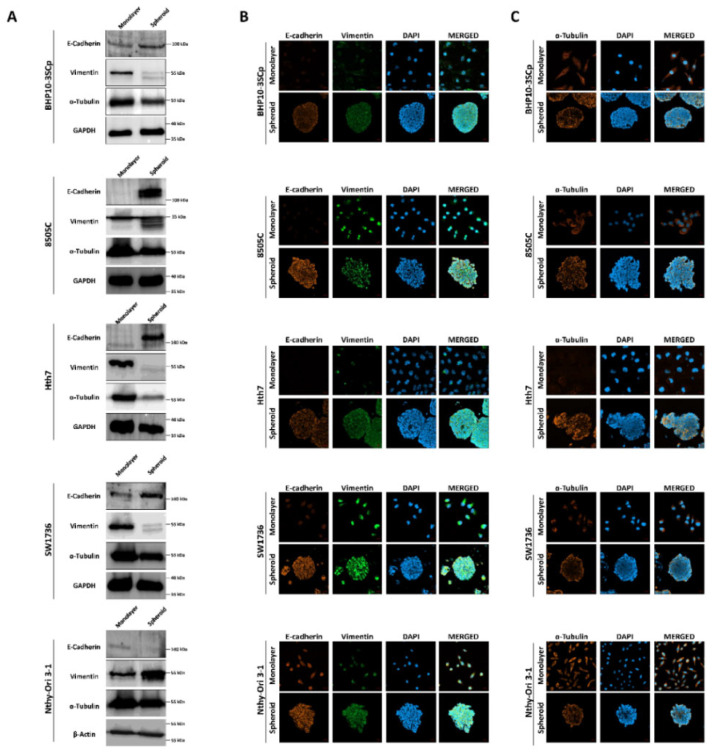
Observation of the extracellular matrix (ECM) and the cytoskeleton under the 2D monolayer and 3D spheroid conditions. E-cadherin and vimentin were used as ECM markers. α-tubulin was used as a cytoskeleton marker. (**A**) Western blot analysis showed E-cadherin, vimentin, and α-tubulin expression. GAPDH and β-Actin were used as loading control. Representative immunofluorescence images showing (**B**) ECM and (**C**) cytoskeleton. Scale bar: 50 µm.

Next, we sought to observe the change in ECM and cytoskeleton components between the 2D monolayer and 3D spheroid cultures. As shown in Figure 6A, the 3D spheroid culture of four thyroid cancer cell lines demonstrated considerably upregulated expression of E-cadherin (an epithelial cell marker) compared to 2D monolayer culture. Quantitative analysis also showed that the 3D spheroid culture significantly induces increases in E-cadherin expression levels (Supplementary Figure S3A–D; BHP10-3SCp: 1.36 ± 0.13, 8505C: 10.55 ± 3.41, Hth7: 9.39 ± 5.68 and SW1736: 2.24 ± 0.68). On immunofluorescence analysis, all thyroid cancer cell lines demonstrated low E-cadherin expression in the 2D monolayer culture (Figure 6B). However, the 3D spheroid culture revealed higher E-cadherin expression, mostly at the outer layer compared to the inner layer. Moreover, vimentin (a mesenchymal cell marker) was expressed in both the 2D monolayer and 3D spheroid cultures (Figure 6B). However, Western blot analysis showed a decreased total amount of vimentin expression, and the protein was cleaved on the 3D spheroid culture environment (Figure 6A). Quantitative analysis with vimentin band intensities revealed increased expression levels in the 3D spheroid culture environment in all thyroid cancer cell lines (Supplementary Figure S3A–D; BHP10-3SCp: 0.22 ± 0.04, 8505C: 0.82 ± 0.10, Hth7: 0.24 ± 0.16 and SW1736: 0.21 ± 0.56). Thyroid follicular epithelial cells demonstrated opposite expression patterns of E-cadherin and vimentin in the aforementioned thyroid cancer cell lines (Figure 6A and Supplementary Figure S3E). Specifically, E-cadherin expression was downregulated (0.65 ± 0.16), whereas vimentin expression was upregulated (2.02 ± 0.60) in the 3D spheroid culture compared to the 2D monolayer culture.

We then compared the expression of α-tubulin (a cytoskeleton marker) between the 2D monolayer and 3D spheroid cultures. Western blot analysis from whole cell lysates showed that α-tubulin expression was slightly downregulated in the 3D spheroid culture compared to the 2D monolayer culture (Figure 6A and Supplementary Figure S3). Additionally, α-tubulin was distinctly expressed in the 2D monolayer culture, regardless of the cell type, mostly localized to the cytoplasm (Figure 6C). However, in the 3D spheroid culture, the outer layer of the spheroid had greater α-tubulin expression than the inner layer.

### 3.8. Change of Proteins Related to Iodide-Metabolizing Machinery under the 2D Monolayer and 3D Spheroid Culture Conditions

**Figure 7:**
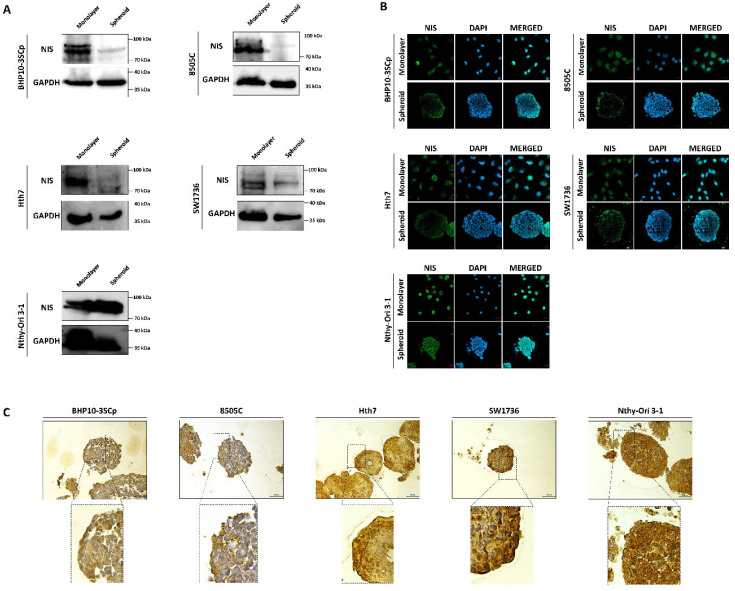
Evaluation of change with sodium iodide symporter (NIS) under 2D monolayer condition and 3D spheroid condition. (**A**) Western blot analysis showed NIS expression. GAPDH was used as a loading control. (**B**) Representative immunofluorescence images showing NIS. Scale bar: 50 µm. (**C**) Representative immunohistochemical images for NIS with spheroids. Scale bar: 50 µm.

**Figure 8:**
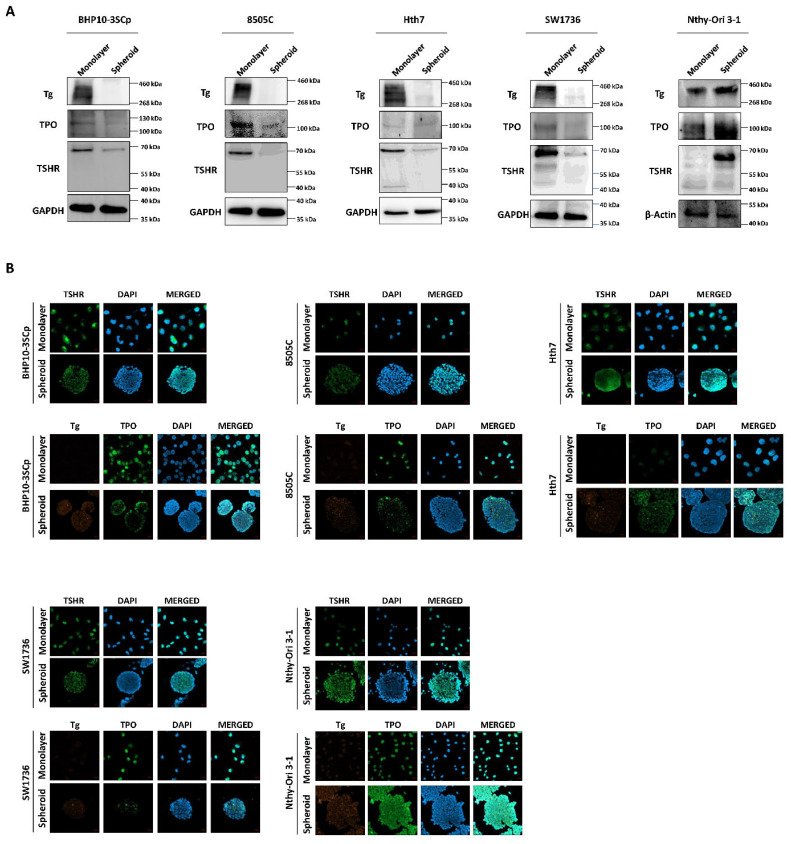
Verification of thyroid-specific proteins expression under 2D monolayer condition and 3D spheroid condition. (**A**) Western blot analysis showing thyroglobulin (Tg), thyroid peroxidase (TPO), and TSH-receptor (TSHR) expression. GAPDH and β-Actin were used as loading control. (**B**) Representative immunofluorescence images showing Tg, TPO, and TSHR. Scale bar: 50 µm.

**Figure 9:**
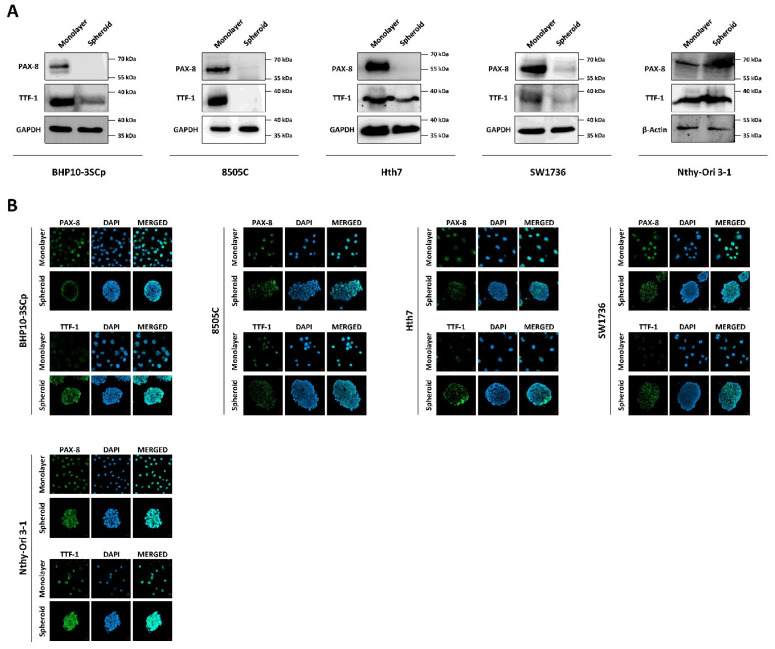
Monitoring of thyroid transcription factors (PAX-8, TTF-1) under the 2D monolayer and 3D spheroid conditions. (**A**) Western blot analysis showing paired box gene-8 (PAX-8) and thyroid transcription factor-1 (TTF-1) expression. GAPDH and β-Actin were used as loading control. (**B**) Representative immunofluorescence images showing PAX-8 and TTF-1. Scale bar: 50 µm.

Both iodide intake capability and iodide concentration are prerequisites for successful RAI therapy [3]. This treatment utilizes the functions of iodide-metabolizing mechanism of thyroid-specific proteins, such as the NIS, thyroid peroxidase (TPO), thyroid-stimulating hormone receptor (TSHR), and thyroglobulin (Tg) along with thyroid transcription factors (paired box gene-8 (PAX-8) and thyroid transcription factor-1 (TTF-1)) [23]. Therefore, we conducted a comparative study about the expression of thyroid-specific proteins and thyroid transcription factors between the 2D monolayer and 3D spheroid culture environment. First, we examined the changes in NIS expression and localization via Western blot analysis. As shown in Figure 7A, the 3D spheroid culture with thyroid cancer cell lines tended to have diminished NIS expression compared to the 2D monolayer culture environment. NIS localization was also predominantly observed in the outer layer, while being low on the inner layer (Figure 7B,C). Contrastively, Nthy-Ori 3-1, as a thyroid follicular epithelial cell line, was not influenced by 3D culture conditions for the decline of NIS expression in Western blot analysis (Figure 7A). Additionally, NIS was uniformly expressed at both the inner and outer layers, unlike in some thyroid cancer cell lines (Figure 7B,C). We also observed changes in other thyroid-specific proteins, including Tg, TPO, and TSHR. Even though each cell line expressed different degrees of thyroid-specific proteins, all cell lines under the 2D monolayer culture demonstrated the expression of those proteins (Figure 8). However, this expression was sharply decreased under the 3D spheroid culture condition (Figure 8A). In the 3D spheroid culture of thyroid cancer cell lines, the expression of thyroid-specific proteins was higher in the outer layer versus the inner layer (Figure 8B). However, the 3D spheroid culture of thyroid follicular epithelial cells showed an even expression of thyroid-specific proteins across both inner and outer layers of spheroids. Thyroid transcription factors, such as PAX-8 and TTF-1, also showed an expression pattern similar to that of thyroid-specific proteins (Figure 9). Thyroid cancer cell lines had lower expression of transcription factors on Western blot analysis in the 3D spheroid culture environment than in the 2D monolayer culture (Figure 9A). On immunofluorescence analysis of thyroid cancer cell lines under 3D spheroid culture, transcription factors were more predominantly expressed at the outer layer compared to the inner layer. However, thyroid follicular epithelial cells had well-expressed transcription factors regardless of the culture environment. Thus, the 3D spheroid culture environment influenced a declined expression of proteins related to iodide-metabolizing mechanisms in thyroid cancer cells, whereas thyroid follicular epithelial cells under this environment were unaffected.

## 4. Discussion

Despite the significant funding of many cancer studies, drug attrition rates are much higher in these cases than other therapeutic fields [24,25]. Merely 5% of drug candidates with an anti-tumor effect in preclinical development are licensed after demonstrating sufficient efficacy with clinical trials in phase III testing [25]. Therefore, most advanced stage metastatic and/or recurrent tumors remain incurable after failure of treatment [6,24]. Several reasons have contributed to the poor effectiveness of anti-cancer drug development, such as insufficient translational research and a lack of adequate preclinical models that recreate the complexity and heterogeneity of cancerous lesions [6,26]. Additionally, most drug development research has focused on cellular proliferation or angiogenesis, while other molecular signaling pathways or targets have been relatively neglected [6]. The 2D monolayer culture method is cost effective, simple to culture, easy to maintain, and can easily be adapted by high-throughput screening for target drugs [14]. Additionally, the cells under the 2D monolayer culture are directly in contact with the microenvironment and have maximal exposure to available nutrients and growth factors [14,27,28]. However, these factors do not sufficiently replicate the conditions of an in vivo tumor with heterogeneous characteristics due to poor cell–ECM interactions, lack of physiological effects against external stimulation, and the absence of drug penetration and drug resistance [14,29]. Thus, these features of the 2D monolayer culture do not recapitulate the tumor microenvironment and architecture, which can lead to misleading and unexpected results for in vivo studies. Conversely, the 3D spheroid culture is more complex, but has a key advantage in its ability to recapitulate the tumor microenvironment and architecture due to the following reasons: First, the 3D spheroid culture system is based on the physiological manner of culturing cells. Second, it maintains structural integrity, allowing for bio-mimicking to occur. Third, there is constant crosstalk with other cells, such as stroma cells, fibroblast cells, immune cells, and endothelial cells [7,14]. Additionally, this is more suitable for investigating drug sensitivity, penetration, and bioavailability because its ECM barriers influence resistance to drug penetration by diffusion, similar to tumor cells in vivo [7,14,30]. Therefore, in understanding the tumor microenvironment, it would be helpful to accurately recapitulate the architecture and biological features of human tumors via 3D spheroid culture before conducting in vivo experiments.

Up to date, both preclinical and clinical trials by multiple researchers have investigated the efficacy of various targets and drugs as a redifferentiation strategy for overcoming RAI-refractory thyroid cancer [31]. However, no clinical trials could anticipate the pharmacologic effects that cause redifferentiation. Various factors may have limited the ability of current studies to develop innovative drugs for converting RAI-refractory thyroid cancers to RAI-sensitive ones. For example, this could be attributed to the 2D monolayer culture, which poorly reflects the 3D architectures observed in natural cancer lesions of various histological origins [32]. Based on previous studies, we conducted a comparative study about the changes of factors associated with iodide-metabolizing machinery under 2D and 3D culture environments in this study. We successfully established 3D culture condition to form MCSs using 1% agarose gel. We excavated 5 of 10 cell lines (four thyroid cancer cell lines and one thyroid follicular epithelial cell line), which showed well-established spheroids under 1% agarose-coated plates and performed further experimentation on these. Importantly, we confirmed that the spheroid formation of thyroid cancers does not rely on characteristics of cell types. Furthermore, the cell number could have caused variations in the degree of cell aggregation. Therefore, the degree of aggregation for spheroid formation would also likely vary due to each cell line having variable characteristics. There are various methods for 3D spheroid generation, such as ultra-low attachment plates, hanging drop, matrix-on-top/embedded and microfluidic device application, and so on [7], and we selected the matrix-on-top method with agarose. In the matrix-on-top method, either agarose or Matrigel are usually used to establish 3D spheroids. Matrigel, which derives from a basement membrane isolated from a murine tumor, is widely used, especially as an invasion assay, tumor xenograft models as well as 3D spheroid culture in preclinical cancer research [33]. It is enriched in laminin, collagen, entactin and soluble growth factors such as fibroblast growth factor (FGF), epidermal growth factor (EGF), transformative growth factor-β (TGF-β), and matrix metalloproteinases (MMP), including MMP-2 and MMP-9 [33,34]. Therefore, the presence of these factors may affect the results of studies either positively or negatively [33]. Although Matrigel does not require specialized equipment, it is difficult to handle when it is in a refrigerated liquid state. In contrast, agarose is cost-effective, no requirement of specialized equipment, high reproducibility, possibility of large-scale production, and thermo-reversible gelation behavior can easy accessibility to establish 3D spheroids [35]. In addition, it is possible to adjust the agarose concentration with different stiffness depending on the purpose of the study. Because agarose is highly permeable to gas and small biomolecules, it can be applied to studies for assessing efficiency of anti-cancer candidates [35,36]. However, there are some drawbacks such that the cell culture depends on the matrix condition, and spheroid sizes and shapes can be variable [22].

Until now, there have been a few precedent studies associated with thyroid differentiation under 3D culture environment [17,19,37]. One article confirmed the different biological and molecular characteristics of thyrospheres compared to human normal and malignant thyroid tissues [19]. Normal thyrospheres had regular shape and were well differentiated; TSH stimulation also maximizes the expression of differentiation markers with downregulation of cancer stem cell markers. Conversely, papillary thyroid cancer (PTC) spheres had irregular shapes, downregulated expression of differentiation markers, and weak TSH-stimulated cAMP production and thyroglobulin expression. However, these reports did not conduct a comparative analysis of 2D monolayer and 3D spheroid culture conditions. Unlike previous studies, we demonstrated that thyroid cancer cells under the 3D spheroid culture environment had downregulated thyroid differentiation markers compared to those in 2D culture. However, thyroid follicular epithelial cells under the 3D spheroid and 2D culture conditions showed a similar expression pattern of thyroid differentiation markers.

After the 3D spheroids were established successfully, we compared the changes in the ECM (including E-cadherin and vimentin) between the 2D monolayer culture and 3D spheroid culture. E-cadherin, as an epithelial marker, was more predominant than vimentin in thyroid cancer cell lines under 3D spheroid culture condition. Conversely, thyroid follicular epithelial cells under the 3D culture condition had downregulated E-cadherin and upregulated vimentin expression. The ECM contributes to cellular structure and function, such as adhesion, migration, proliferation, and differentiation [38]. E-cadherin is closely associated with cell-to-cell adhesion, and its upregulation indicates a correlation between stemness and compactness in 3D spheroid structure. Some reports have shown that cell lines with more epithelial characteristics produce more aggregation versus regular cell lines [39,40]. Melissaridou et al. reported that the establishment of stable spheroids with compact condensation using head and neck squamous cell carcinoma induced the downregulation of epithelial–mesenchymal transition (EMT)-related genes, with decreased N-cadherin and vimentin expression [40]. EMT and reverse MET (mesenchymal–epithelial transition) are considerably involved in stemness balance in spheroids regardless of cell type [41]. However, α-tubulin, a constituent of microtubules among cytoskeletons, is involved in the maintenance of cell morphology, migration, and intracellular transport through the process of polymerization. Loose cytoskeleton tension accompanies the morphology of compacted cells with reduced size. In our study, we confirmed decreased α-tubulin expression levels under the 3D spheroid culture condition compared to the 2D monolayer culture.

In this study, the spheroid formation, regardless of the cell type, induced a decrease in cell proliferation and ATP levels and an increase in HIF-1α. Under the 3D spheroid culture environment, cells have a three-layered structure, including the proliferating zone (outer layer), quiescent viable zone (intermediate layer), and necrotic zone (inner layer) [42]. These heterogeneous cell layers are defined due to the diffusion of oxygen and nutrients into the spheroids [43]. Marked proliferation is seen in the outer layer due to the sufficient supply of nutrients and oxygen from the culture medium. However, toward the inner layer, nutrients and oxygen become more insufficient, decreasing the proliferation capacity [43]. After the formation of spheroids, the inner layer receives only a limited supply of oxygen and nutrients from the outside via diffusion. Thus, this mechanism is behind the decreased cell proliferation and ATP levels under the 3D culture condition versus the 2D monolayer culture in all cell lines.

Oxygen deficiency triggers changes in gene expression through the induction of hypoxia factors, such as HIF-1α. The promotion of HIF-1α under a hypoxic environment has various functions involving drug efflux, apoptosis, autophagy, DNA damage, mitochondrial activity, and p53 [44]. Additionally, hypoxia reduces pH, leading to an acidic tumor microenvironment [44]. Consequently, these mechanisms become a major cause for drug resistance and eventual therapy failure. Several reports have demonstrated the influence of a hypoxic environment to drug response [44,45,46,47]. Qin et al. demonstrated that a hypoxic environment interrupts the growth-inhibitory effect of vemurafenib by upregulating the HGF–MET signaling pathway in melanomas with *BRAF^V600E^* mutation [45]. An article reported that the *BRAF^V600E^* mutation, which occurs in ~45% of patients with PTC, affects HIF1-α expression in thyroid cancers by regulating hypoxia and the *BRAF^V600E^*-mediated signaling pathway [48]. Zerilli et al. suggested a strategy for overcoming upregulated HIF1-α expression by administering sorafenib, which could block *BRAF^V600E^* and its downstream in *BRAF^V600E^*-mutated thyroid cancers [49]. In the aforementioned reports, oxygen-independent regulation trigger increased HIF1-α expression, suggesting that HIF1-α expression could be induced by growth factor signaling pathways, such as MAPK and PI3K-Akt. Several reports have associated hypoxia with thyroid cancer progression under oxygen-dependent regulation [48,49,50,51,52]. Burrows et al. reported that HIF1-α was expressed in all thyroid tumor types but not in normal tissue [48]. They demonstrated that HIF1-α expression was higher in ATC, which showed undifferentiated characteristics compared to other tumor types, such as PTC and follicular thyroid cancer (FTC). Furthermore, they reported that the highest expression of HIF1-α and CA-9 had a greater number of spontaneous metastatic colonies to lung tissues [52]. Collectively, these reports suggest that HIF1-α is closely related to the pathophysiology of thyroid cancer, specifically in terms of tumor aggressiveness, progression, and metastasis with characteristics of dedifferentiation [48,52]. Several articles have reported the relationship between hypoxia and NIS expression [21,53,54]. Lan et al. demonstrated that the overexpression of HIF1-α in thyroid cancers decreased NIS expression [53]. Moreover, another article reported that the tumor microenvironment, characterized by hypoxia and quiescence, could affect NIS expression and RAI uptake in exogenous NIS-expressing cancer cells [21]. Therefore, we speculated that 3D spheroid culture is hypoxic due to increased HIF-1α expression and that this phenomenon could influence the decline in thyroid-specific proteins and thyroid transcription factors. However, further studies are needed to investigate the other factors involved in the decreased expression of thyroid-specific proteins and thyroid transcription factors because the patterns of various elements also change between the 2D monolayer and 3D spheroid culture environments.

## 5. Conclusions

The 3D spheroid culture environment is similar to the in vivo condition because it alters numerous cellular and functional activities, including morphology, cellular proliferation, viability, hypoxia, ECM, cytoskeleton, and thyroid differentiation, compared to the conventional 2D monolayer culture environment. In vitro experiments using the 3D spheroid culture can speed up the discovery of new drugs.

## Data Availability

Data will be made available upon reasonable request to corresponding author.

## Supplementary Materials

The following supporting information can be downloaded at: https://www.mdpi.com/article/10.3390/cells11223559/s1, Figure S1: Establishment of 3D spheroid with papillary thyroid cancer cell lines; Figure S2: Establishment of 3D spheroid with several thyroid cells; Figure S3: Quantitative analysis of extracellular matrix (ECM) and cytoskeleton proteins expression; Scheme S1: List of used antibodies for western blot analysis; Scheme S2: List of used antibodies for immunofluorescence analysis
