# Supplementary material for: Different Expression of Thyroid-Specific Proteins in Thyroid Cancer Cells between 2-Dimensional (2D) and 3-Dimensional (3D) Culture Environment
Source: Cells. 2022 Nov 10;11(22):3559. doi: 10.3390/cells11223559 (PMC9688357; doi:10.3390/cells11223559)
Supplement: Supplementary file 1 [file cells-11-03559-s001.zip › cells-1955279-supplementary/[Final] Scheme.pdf]

Scheme S1. List of used antibodies for western blot analysis

Primary Antibodies				Secondary Antibodies	
**Name**	**Dilution Ratio**	**Catalog Number**	**Manufacturer**	**Name**	**Dilution Ratio**
NIS	1:4000	MS-1653	Thermo Fisher	Anti-Mouse IgG, HRP-linked Antibody	1:8000
PAX-8	1:1000	SC-81353	Santa Cruz	Anti-Mouse IgG, HRP-linked Antibody	1:2000
TTF-1	1:1000	SC-53136	Santa Cruz	Anti-Mouse IgG, HRP-linked Antibody	1:2000
Thyroperoxidase (TPO)	1:1000	SC-58432	Santa Cruz	Anti-Mouse IgG, HRP-linked Antibody	1:2000
TSH-receptor (TSHR)	1:1000	SC-515556	Santa Cruz	Anti-Mouse IgG, HRP-linked Antibody	1:2000
Thyroglobulin (Tg)	1:1000	SC-365997	Santa Cruz	Anti-Mouse IgG, HRP-linked Antibody	1:2000
E-cadherin	1:5000	SC-7870	Santa Cruz	Anti-Rabbit IgG, HRP-linked Antibody	1:10000
Vimentin	1:5000	SC-66002	Santa Cruz	Anti-Mouse IgG, HRP-linked Antibody	1:10000
α-Tubulin	1:5000	#2125	Cell Signaling	Anti-Rabbit IgG, HRP-linked Antibody	1:10000
PCNA	1:5000	#13110	Cell Signaling	Anti-Rabbit IgG, HRP-linked Antibody	1:10000
HIF1-α	1:1000	#36169	Cell Signaling	Anti-Rabbit IgG, HRP-linked Antibody	1:2000
GAPDH	1:5000	sc-47724	Santa Cruz	Anti-Mouse IgG, HRP-linked Antibody	1:10000
β-Actin	1:5000	#4970	Cell Signaling	Anti-Rabbit IgG, HRP-linked Antibody	1:10000

Scheme S2. List of used antibodies for immunofluorescence analysis

Primary Antibodies				Secondary Antibodies	
**Name**	**Dilution Ratio**	**Catalog Number**	**Manufacturer**	**Name**	**Dilution Ratio**
NIS	1:250	ab17795	Abcam	Anti-mouse IgG (H+L), F(ab')2 Fragment (Alexa Fluor® 488 Conjugate)	1:500
PAX-8	1:250	SC-81353	Santa Cruz	Anti-mouse IgG (H+L), F(ab')2 Fragment (Alexa Fluor® 488 Conjugate)	1:500
TTF-1	1:250	SC-53136	Santa Cruz	Anti-mouse IgG (H+L), F(ab')2 Fragment (Alexa Fluor® 488 Conjugate)	1:500
TPO	1:100	SC-58432	Santa Cruz	Anti-mouse IgG (H+L), F(ab')2 Fragment (Alexa Fluor® 488 Conjugate)	1:200
TSHR	1:250	SC-515556	Santa Cruz	Anti-mouse IgG (H+L), F(ab')2 Fragment (Alexa Fluor® 488 Conjugate)	1:500
Tg	1:100	ab156008	Abcam	Anti-rabbit IgG (H+L), F(ab')2 Fragment (Alexa Fluor® 555 Conjugate)	1:200
E-cadherin	1:250	SC-7870	Santa Cruz	Anti-rabbit IgG (H+L), F(ab')2 Fragment (Alexa Fluor® 555 Conjugate)	1:500
Vimentin	1:250	SC-66002	Santa Cruz	Anti-mouse IgG (H+L), F(ab')2 Fragment (Alexa Fluor® 488 Conjugate)	1:500
α-Tubulin	1:250	#2125	Cell Signaling	Anti-rabbit IgG (H+L), F(ab')2 Fragment (Alexa Fluor® 555 Conjugate)	1:500
PCNA	1:250	#13110	Cell Signaling	Anti-rabbit IgG (H+L), F(ab')2 Fragment (Alexa Fluor® 555 Conjugate)	1:500
Ki-67	1:250	SC-23900	Santa Cruz	Anti-mouse IgG (H+L), F(ab')2 Fragment (Alexa Fluor® 488 Conjugate)	1:500
