# Supplementary material for: Different Expression of Thyroid-Specific Proteins in Thyroid Cancer Cells between 2-Dimensional (2D) and 3-Dimensional (3D) Culture Environment
Source: Cells. 2022 Nov 10;11(22):3559. doi: 10.3390/cells11223559 (PMC9688357; doi:10.3390/cells11223559)
Supplement: Supplementary file 1 [file cells-11-03559-s001.zip › cells-1955279-supplementary/[Final] Supplementary Data_3D culture.pdf]

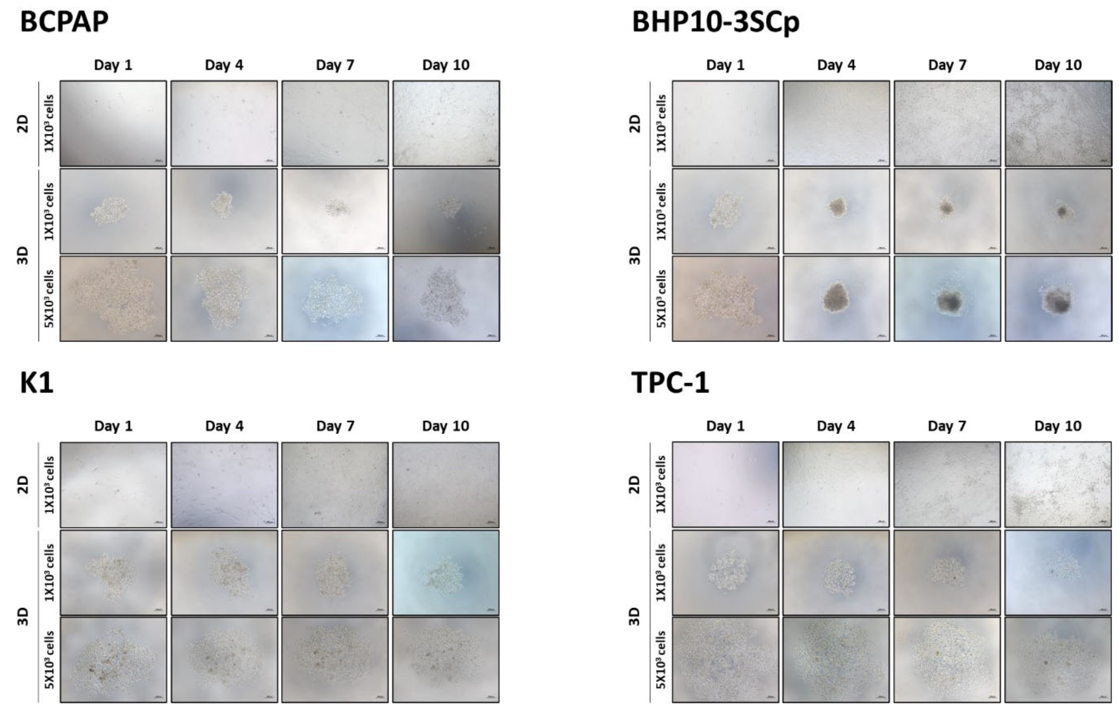

Supplementary Figure 1. Establishment of 3D spheroid with papillary thyroid cancer cell lines. Four papillary thyroid cancer lines tried to format the 3D spheroid using 1% agarose-coated plates. The formation of spheroids was observed with contrast microscopy. Scale bar: 50 µm

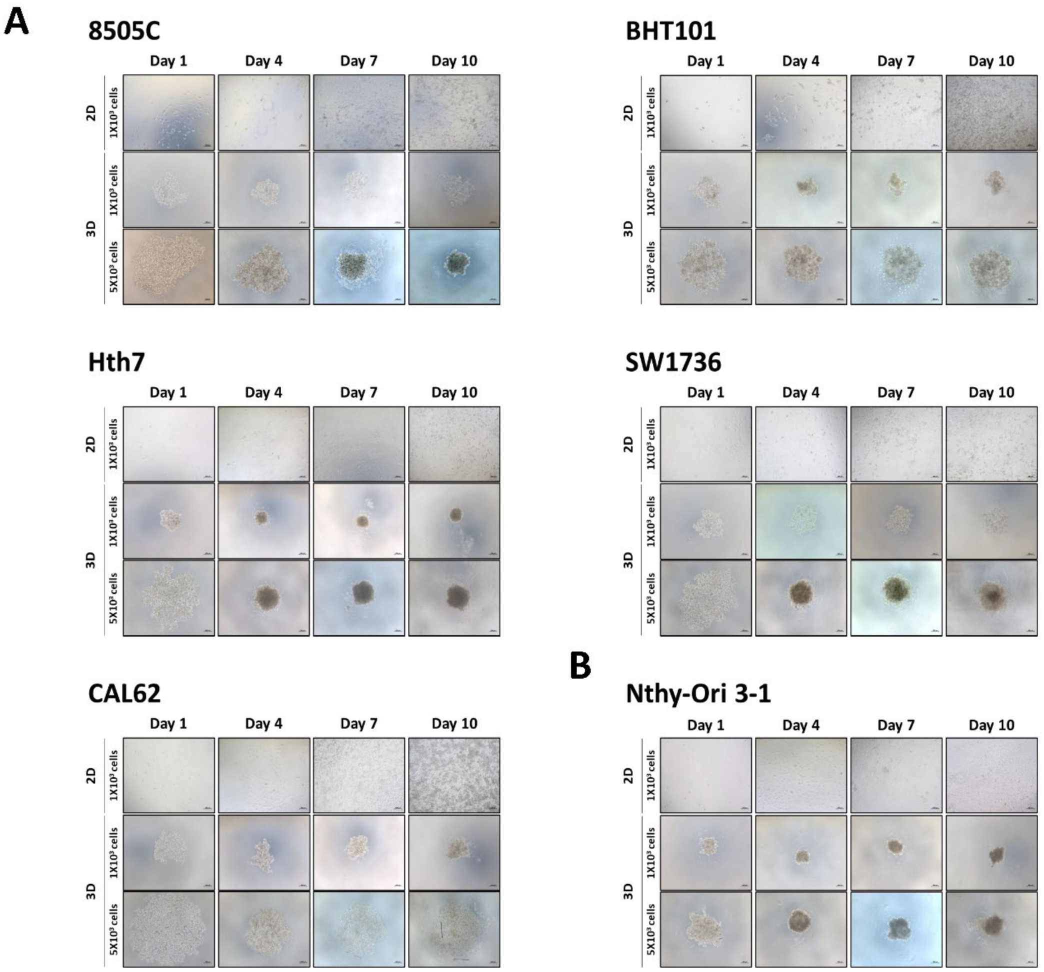

Supplementary Figure 2. Establishment of 3D spheroid with several thyroid cells. The five anaplastic thyroid cancer cell lines and one thyroid follicular epithelial cell line tried to format the 3D spheroid using 1% agarose-coated plates. The formation of spheroids was observed with contrast microscopy. (A) Anaplastic thyroid cancer cell lines. (B) Thyroid follicular epithelial cell line. Scale bar: 50 µm

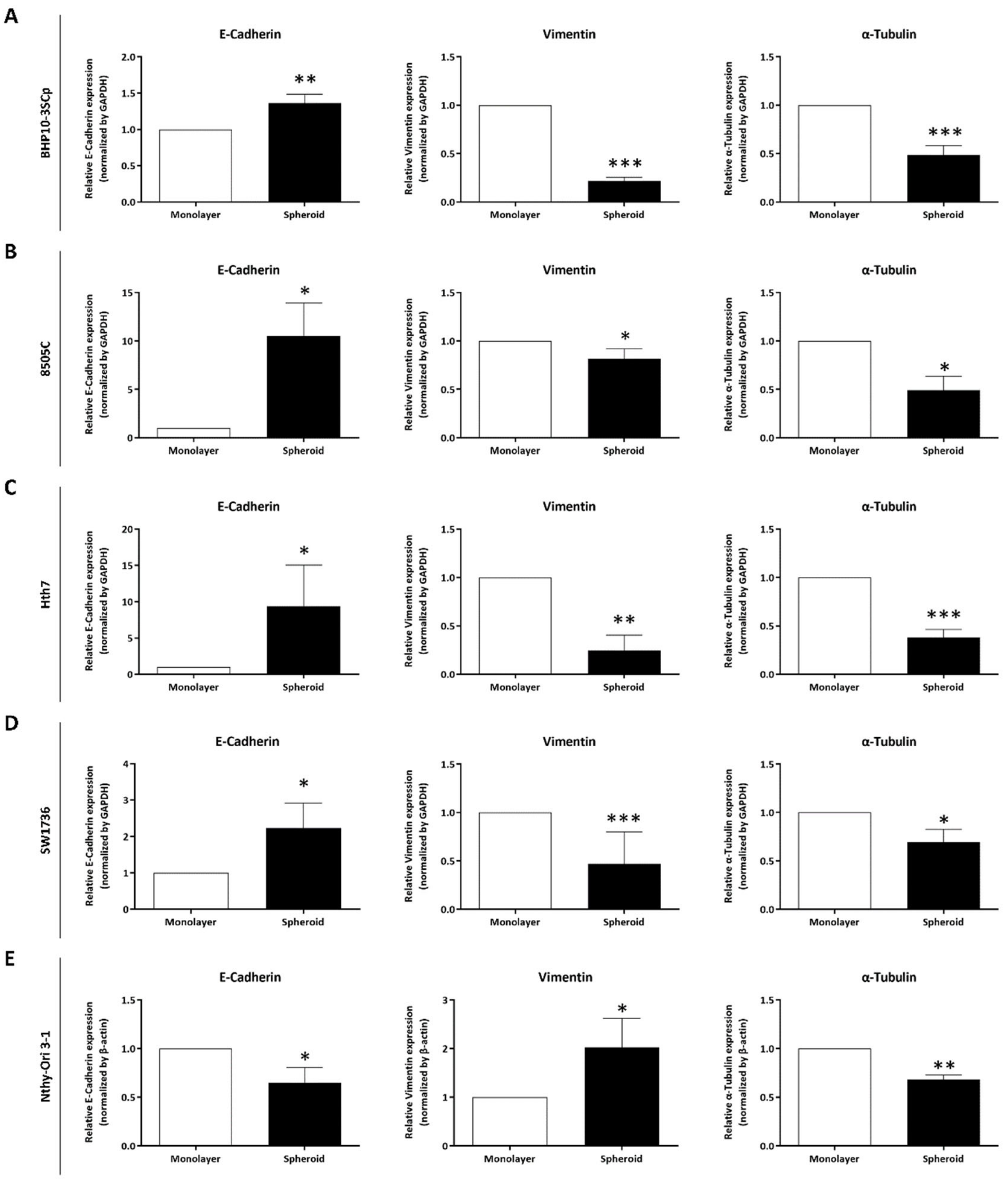

Supplementary Figure 3. Quantitative analysis of extracellular matrix (ECM) and cytoskeleton proteins expression. Band intensities with E-Cadherin, vimentin and α-tubulin in western blot images were quantified. GAPDH and β-Actin were used as loading control. The mean ± standard deviation (SD) values from at least three independent experiments are presented. *$p < 0.05$, **$p < 0.01$ and ***$p < 0.001$ (Student's *t*-test).
